# A Rare Case Report of Neurodegenerative Disease With Oro-Dental Trauma

**DOI:** 10.7759/cureus.81450

**Published:** 2025-03-30

**Authors:** Sudipa Ghosh, Pramod G Vittobarao, Shivaprasad S, Ashok L, Shambulingappa P

**Affiliations:** 1 Oral Medicine and Radiology, Bapuji Dental College and Hospital, Davangere, IND

**Keywords:** autosomal recessive, batten disease, late infantile, neuronal ceroid lipofuscinoses, traumatic tooth

## Abstract

Batten disease, also known as Spielmeyer-Vogt-Sjögren-Batten disease, is a rare and deadly autosomal recessive neurodegenerative disease. It is the most prevalent type among a group of diseases known as neuronal ceroid lipofuscinoses (NCLs).

The patient, a 31-year-old woman, presented to the Oral Medicine and Radiology Department with the primary complaint of knocking out her upper front teeth after she had suffered a seizure and fell out of bed onto the floor. She has Batten disease, according to her medical history (NCL2). The diagnosis and treatment of an oro-dental issue are presented in this instance.

As an oral physician, understanding Batten disease is crucial because it requires a multidisciplinary approach to manage the oral health issues associated with it. Early intervention and continuous support are essential to maintaining the well-being of affected individuals.

## Introduction

A class of debilitating and fatal neurodegenerative lysosomal storage diseases that primarily affect children is known as neuronal ceroid lipofuscinoses (NCLs). Even though NCLs are uncommon conditions on their own, when combined, they make up the most prevalent neurodegenerative illness in kids [1]. The cell elimination of a squander product known as ceroid lipofuscin is hampered by these disorders. Ceroid lipofuscin accumulation within cells is a sign of lysosomal dysfunction [2]. Even so, Batten disease was formerly thought to be a juvenile form of NCL. In honor of Frederick Eustace Batten, an English pediatrician and neurologist known as the father of pediatric neurology, the illness bears his name [3]. The global incidences are approximately one in 100,000 live births. The highest occurrence of NCL disease is found in Scandinavian countries, including Finland [4]. Individuals with Batten disease often experience swallowing and feeding difficulties. Swallowing problems will occur and worsen until the oral feed does not meet nutritional requirements, and there is a high risk of aspiration, poor oral hygiene, biofilm around the teeth, and gingivitis. Cardiorespiratory failure and sepsis secondary to aspiration pneumonia are frequent in these infants, so control of the secretion is critical [5]. Additionally, they may have dry mouth, bruxism, and avulsed teeth that can further complicate their dental care. This essay's goal is to shed more light on oro-dental trauma with neurodegenerative diseases by describing one such exceptional illustration.

## Case presentation

The patient, a 31-year-old woman, came to the Oral Medicine and Radiology Department with the chief complaint that her upper front teeth had been knocked out after she fell out of bed onto the floor six days ago due to a seizure attack. Within an hour of the incident, she went straight to the dentist, and the knocked-out tooth was reinserted. The parents of the patient informed us that it was not the first scenario due to epilepsy. She was born from a non-consanguineous marriage with normal full term and normal perinatal period. She was vaccinated appropriately for her age, and her developmental progress was normal. 

The patient reports that she has had seizures since she was four years old, has had a fever every 15-20 days, and has lost interest in studies and limb coordination since she was seven years old. The language was typical. Horizontal nystagmus was present, and the muscle tone of all four limbs was reduced, as was the broad base, the regular step, the lateral swaying, and the typical arm swing, suggestive of myopathic gait. After being diagnosed with progressive myoclonic epilepsy at the age of 13, she received drug therapy. 

The medical examination records such as electroencephalogram (EEG) are abnormal and showed generalized frontal dominant epileptiform discharges. Computed tomography (CT) and magnetic resonance imaging (MRI) revealed cerebellar atrophy. No optic atrophy and no Lafora bodies were found in the skin biopsy. The overall assessment suggests that she most likely suffered from neuronal dysfunction. She underwent medicine therapy and physiotherapy, such as spine exercise. After five years, she was suddenly paralyzed for nine years. Again, she started activities one year ago, and she is currently under treatment. Family history indicates that the patient's father is known to have suffered from progressive relapsing multiple sclerosis for eight years.

On general examination, the patient was conscious, oriented, and afebrile, with a pulse rate of 80 beats per minute, a respiratory rate of 17 cycles per minute, and a blood pressure of 120/80 mmHg. She showed no symptoms of pallor, jaundice-cyanosis, or clubbing. There was poor coordination of proximal phalanges as well as reduced tenderness when standing unsupported on one foot. Hyperreflexia of the lower limb, slurred speech, slow learning, echolalia, and difficulty in writing were noted.

On extraoral examination, there was no gross facial asymmetry or scar on the lip. The bilateral group of submandibular lymph nodes was small, oval-shaped, smooth, soft in consistency, non-tender, and not attached to the underlying structure on palpation. Examination of the temporomandibular joint, mouth opening, and bilateral symmetrical jaw movement were all found to be normal.

**Figure 1:**
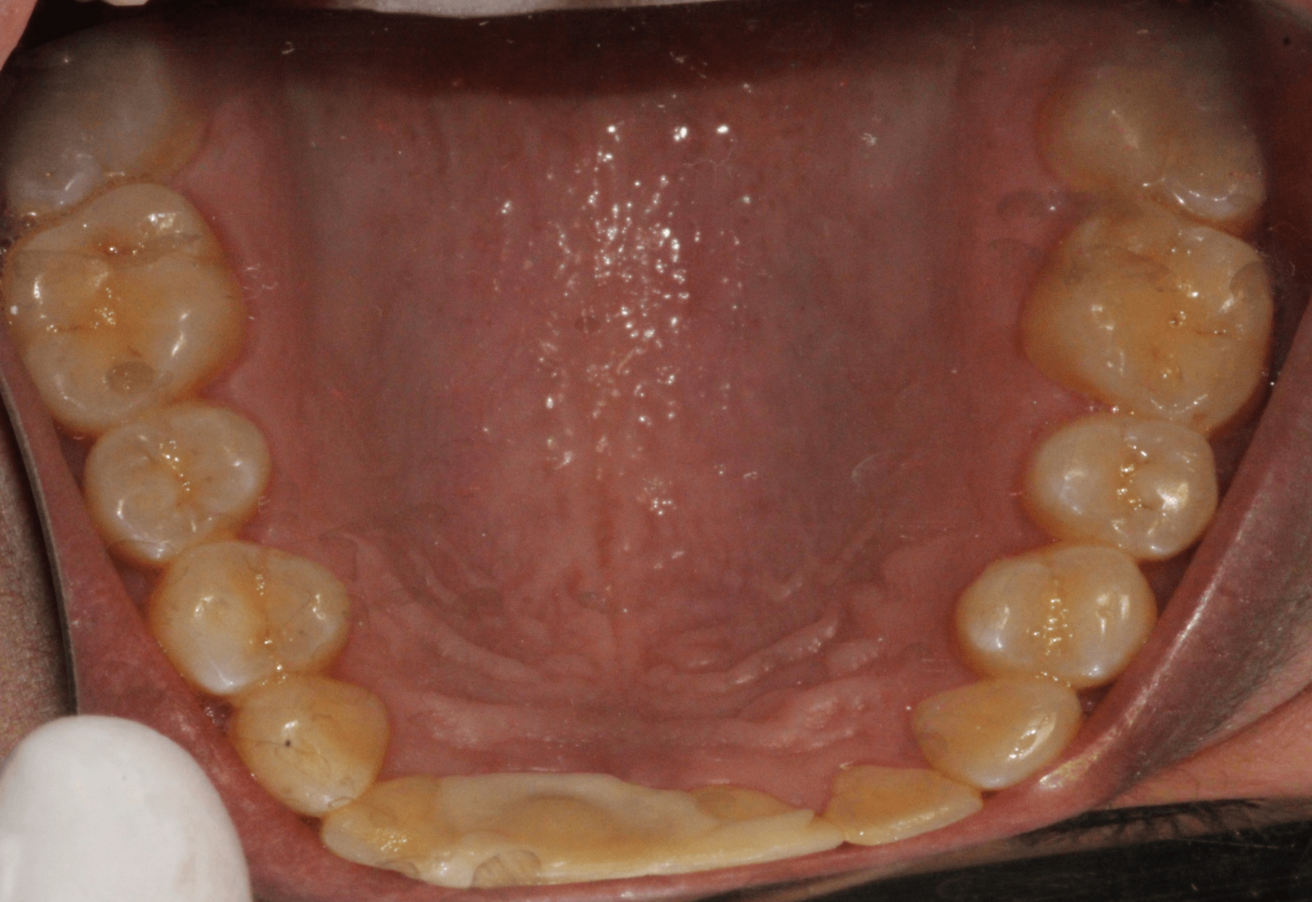
Maxillary arch and palatal splint in the right permanent maxillary central and lateral incisors

**Figure 2:**
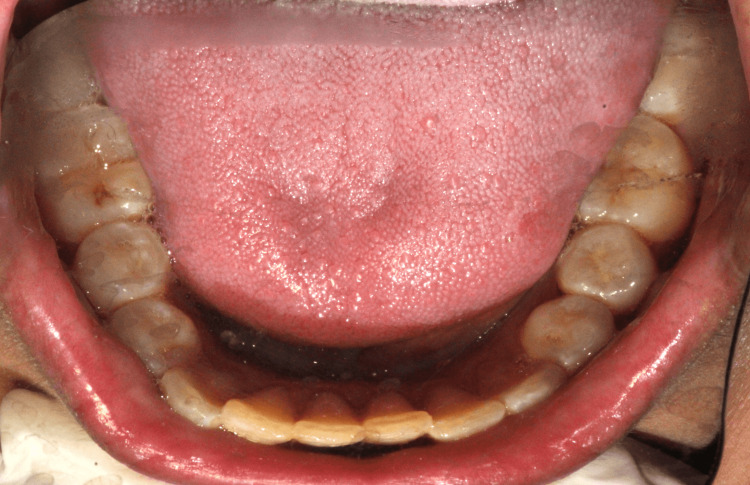
Mandibular arch

On intraoral examination, the masticatory, lining, and specialized mucosa appeared normal. No lacerations to the soft tissue were observed. Generalized, soft, and edematous marginal gingiva was present. There was a normal complement of the permanent dentition except the third molars (Figure 1 and Figure 2).

**Figure 3:**
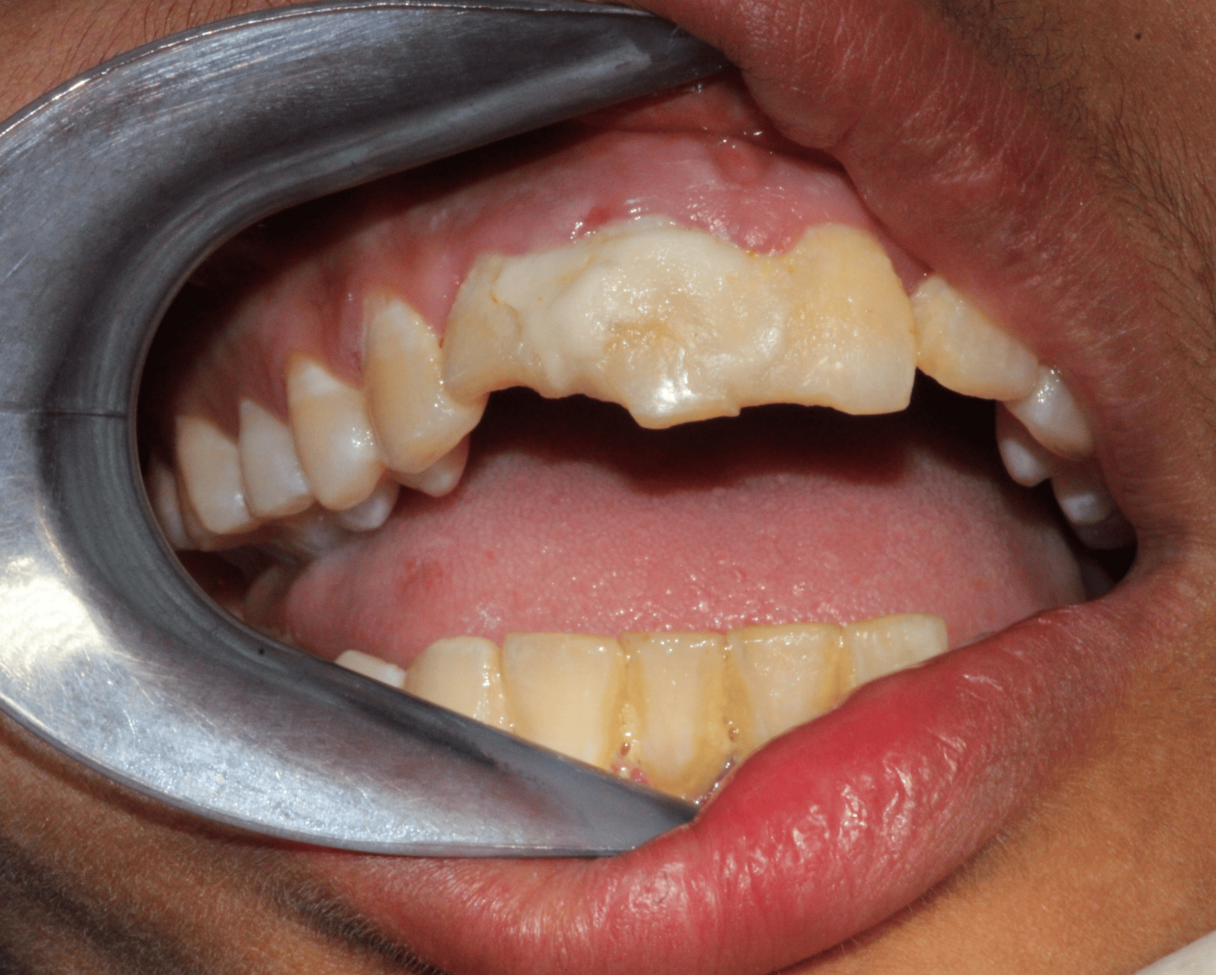
Intraoral photograph (reinserted tooth with splint in the right maxillary central and lateral incisors and Ellis class II fracture)

On percussion, teeth were tender, and vestibular tenderness was noted in relation to 11, 12, and 21. Splint with respect to 11 and 12 was done using a resin-modified dental material (Figure 3). The working diagnoses were Ellis class II fracture with respect to 11 and 12 and chronic generalized gingivitis.

**Figure 4:**
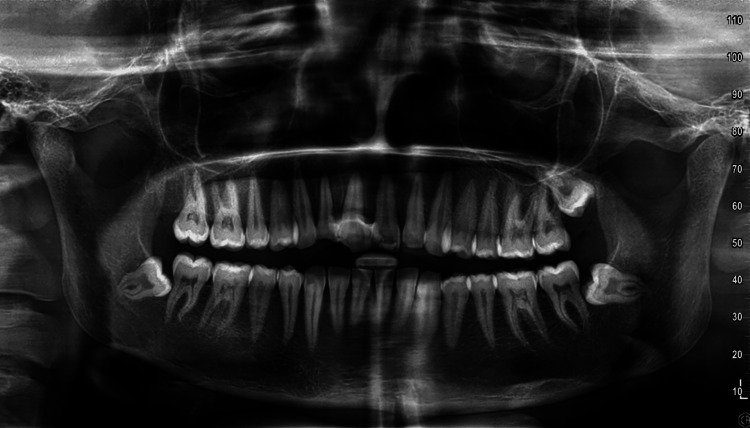
Preoperative orthopantomogram

The intraoral periapical radiograph revealed loss of lamina dura and expansion of the periodontal ligament space in the apical one-third of the root with respect to 12, suggestive of acute apical periodontitis of 12. Additionally, the orthopantomogram showed mesio-angular impaction with respect to 48, disto-angular impaction with respect to 28, and horizontal impaction with respect to 38 (Figure 4).

The patient underwent oral prophylaxis with a splint after systemic and psychological evaluation and was kept under observation and recalled for follow-up. At the second appointment, she showed the reports of the Institute of Human Genetics-Genetics Center of India indicating that a lysosomal enzyme study was performed using a 4-methylumbelliferone (4-MU)-specific substrate for tripeptidyl peptidase-1 (TPP-1). β-Galactosidase was used as a reference enzyme and showed reduced TPP-1 activity with normal β-galactosidase enzyme activity. It is suspected that the patient suffers from NCL2 or Batten disease.

## Discussion

Batten disease is an autosomal recessive inherited disorder. It affects men and women equally. It is often misdiagnosed as retinitis pigmentosa, macular degeneration, intellectual disability, epilepsy, attention deficit hyperactivity disorder, autism, and even schizophrenia [6]. 

Although an autoimmune etiology has also been suggested, genetic mutations are the main cause of Batten disease. Different mutations in the genes encoding endolysosomal system proteins are present in each type of NCL. Defects in insoluble transmembrane proteins, cytosolic protein deficiencies, and soluble lysosomal enzyme defects are the three categories into which these can be separated [2]. The endoplasmic reticulum, which is a component of the endolysosomal system, or the lysosomal membrane may contain these transmembrane proteins. 

As a result of lysosome issues, neurons gradually die. Early growth and development in patients with NCLs are usually normal, and they meet developmental milestones at the right times. Nevertheless, once the illness strikes, advancement halts, and the learned abilities deteriorate. The different classifications of the disease by the occurrence of symptoms are as follows: congenital (beginning at birth), infantile (usually beginning within 6-18 months or during the first year of life), late infantile (typically start at 2-4 years old), juvenile onset (beginning between four and 10 years of age or generally between four and seven years of age), and late juvenile onset period (between the ages of eight and 16) and adulthood (starting in late teens to middle adulthood) [6]. 

Widespread clinical features of Batten disease include visual impairment, inability to achieve normal developmental milestones and/or developmental regression, behavioral problems, progressive brain atrophy, seizures, cognitive decline, and dementia [7]. However, the order and frequency of these symptoms vary between the different subtypes and variants for each genetic mutation [8].

The 14 NCL types that are described in the literature serve as the basis for the various representations of NCL. The most common forms are NCL1, NCL2, and NCL3. These characteristics set NCL1 (classic form with infantile onset) apart. The impacted child may experience speech impairment, motor weakness, seizures, and microcephaly. Without causing noticeable alterations to the eyelids, conjunctiva, cornea, iris, or even vitreous humor, the majority of the anterior portion of the eye is usually unaffected by infantile NCL. Typically, intraocular pressure is within normal limits. Retinal pigment epithelium (RPE) atrophy, retinal vascular narrowing, and optic atrophy-related pallor of the optic nerve head can all be seen in the fundus. The macula showed signs of pigment buildup and distinctive "patchy annular defects". Traditionally, patients with NCL1 disease do not exhibit peripheral retinal pigmentation [8].

The majority of patients with NCL2 (classic late infantile-onset form) develop eye symptoms towards the end of the illness, typically at four to five years old. Around the ages of two to three, the kid starts to experience speech delays and epilepsy; by the time they are five, ataxia and global developmental delay may also be present. There have been no reports of anterior segment manifestations in the writings. There may be fulminant bull's-eye maculopathy in the fundus. Patients should have their speech, visual, motor, and epileptic symptoms assessed [8,9].

As regards NCL3 (classic juvenile form), neuropsychiatric symptoms either precede or occur concurrently with visual symptoms. When a patient presents to an ophthalmologist, they are typically between five and eight years old. The child may have eccentric vision or missing vision in addition to rotational nystagmus. A fundus examination may show peripheral pigmentation, arteriolar narrowing, and pallor of the optic nerve head, all of which are signs of normal to severe pigmentary retinopathy. Coats disease is an example of exudative retinal vasculopathy, which can be seen in rare instances. The majority of patients experience behavioral issues, including depressive symptoms, physical aggression, and tantrums. To correctly identify the various forms of this complex neurodegenerative disease, it is essential to consider key diagnostic criteria, symptoms, and accessible techniques for testing [9,10].

Genetically, it is now recognized that NCLs exhibit substantial genotype-phenotype heterogeneity, meaning that distinct mutations in one or more genes can result in identical phenotypes. Similarly, a single gene mutation can cause distinct phenotypes in members of the same family. Chromosome 11p15 has been identified as the location of the genetic defect responsible for the late infantile form (NCL2) [11]. 

Biochemically, TTP-1 levels for NCL2 may be assessed in the saliva, dried blood spots, cultured fibroblasts, and leukocytes. TTP1 activity in NCL2 is less than 4% of the normal, and fibroblasts produce about 17,000 μmol of amino acids per hour per milligram of protein [12,13].

During neuroimaging, such as MR spectroscopy and MRI, NCL reveals only minor abnormalities until early adolescence in NCL3 and predominant cerebellar atrophy over the brain in NCL2. Early on in NCL2 disease, there is noticeable cerebellar atrophy. Degeneration and widespread cortical and subcortical hypometabolism happen quickly in NCL2. This is linked to anatomical neuroimaging that shows rapidly increasing brain atrophy. Several distinct curvilinearly profiled cytoplasmic inclusions usually occur in classic NCL2 disease. Vacuolated lymphocytes can be seen using blood smear microscopy [14,15].

Histological abnormalities come in a wide variety and occasionally contradict each other. A microglial response may be triggered by the early degeneration of dendritic spines in apical dendrites, leading to neuroinflammation. Apart from stimulating microglia, macrophages also phagocytose cell debris and lip pigments. In every type of childhood NCL, the pathological process involves the retina. Retinal atrophy is more obvious in NCL1 and NCL3 but less obvious in NCL2, and our case report appreciates this feature [16].

Treatment for NCL primarily focuses on symptom management, anticonvulsants, antiepileptics, antispasmodics, and supportive therapies like proper sleep hygiene, a balanced diet, and mental and physical therapy. The age at onset is one of the main factors affecting the prognosis [16].

## Conclusions

Neurological disorders affect dental tissues in different ways, and the treatment of oral health in patients with neurological disorders is complex and requires specific care that is reasonably consistent with the occurrence of specific injuries, such as a traumatic dental injury due to a seizure, which is one of the manifestations of Batten disease. Classical conversation between a neurologist and a dentist is necessary to find the best possible answer to a question about the patient. Professional collaboration between dentists, pediatric neurologists, nutritionists, and occupational therapists is essential in order to provide specialist care to facilitate the comfort of patients suffering from debilitating diseases. Healthcare providers should work as a team to plan ahead for this complex world of neurodegenerative diseases. The prevention of and regular examination of dental diseases are all part of good dental care. In treating people with Batten disease, it is vital to put the patient and their family at the center of the treatment. Addressing the patient's and their caregivers' emotional, psychological, and social needs is part of this in addition to their physical symptoms.
